# Biotic formation of methylmercury: A bio–physico–chemical conundrum

**DOI:** 10.1002/lno.11366

**Published:** 2019-11-12

**Authors:** Andrea G. Bravo, Claudia Cosio

**Affiliations:** ^1^ Department of Marine Biology and Oceanography, Institute of Marine Sciences Spanish National Research Council (CSIC) Barcelona Spain; ^2^ Université de Reims Champagne Ardennes, UMR‐I 02 INERIS‐URCA‐ULH SEBIO, Unité Stress Environnementaux et BIOsurveillance des milieux aquatiques Reims France

## Abstract

Mercury (Hg) is a natural and widespread trace metal, but is considered a priority pollutant, particularly its organic form methylmercury (MMHg), because of human's exposure to MMHg through fish consumption. Pioneering studies showed the methylation of divalent Hg (Hg^II^) to MMHg to occur under oxygen‐limited conditions and to depend on the activity of anaerobic microorganisms. Recent studies identified the *hgcAB* gene cluster in microorganisms with the capacity to methylate Hg^II^ and unveiled a much wider range of species and environmental conditions producing MMHg than previously expected. Here, we review the recent knowledge and approaches used to understand Hg^II^‐methylation, microbial biodiversity and activity involved in these processes, and we highlight the current limits for predicting MMHg concentrations in the environment. The available data unveil the fact that Hg^II^ methylation is a bio‐physico‐chemical conundrum in which the efficiency of biological Hg^II^ methylation appears to depend chiefly on Hg^II^ and nutrients availability, the abundance of electron acceptors such as sulfate or iron, the abundance and composition of organic matter as well as the activity and structure of the microbial community. An increased knowledge of the relationship between microbial community composition, physico‐chemical conditions, MMHg production, and demethylation is necessary to predict variability in MMHg concentrations across environments.

## *The mercury problem*

Mercury (Hg) is a natural and ubiquitous trace metal in the environment that might damage the central nervous system and causes tremors, distorted speech, kidney effects, respiratory failure, dizziness, blurred vision, hallucinations, and even death in severely exposed people (Clarkson and Magos 2006). This pollutant is naturally emitted during episodic events such as volcanic eruptions or ubiquitous weathering of Hg‐containing rocks in the Earth's crust and geothermal activity. Among anthropogenic Hg sources, artisanal and small‐scale gold mining, coal combustion, production of nonferrous metals, cement production, and disposal of wastes containing Hg are of special concern (UNEP 2013). Hg is unique among transition metals due to its high volatility as gaseous elemental Hg (Hg^0^), with a residence time in the atmosphere of about 6–12 months, allowing for long‐range transport of Hg. Although both Hg^0^ and inorganic divalent Hg (Hg^II^) are released from many sources through a variety of natural and anthropogenic processes, the reported rise in Hg levels in the biosphere, and in terrestrial and marine systems is a consequence of anthropogenic emissions (Amos et al. 2013; Lamborg et al. 2014; Kocman et al. 2017). In contrast to Hg^0^ and Hg^II^, direct anthropogenic sources of organic Hg, mono‐methylmercury (MMHg, i.e., CH_3_Hg^+^) or dymethylmercury (DMHg, i.e., [CH_3_]_2_Hg) are scarce.

The chemical behaviors of the different chemical forms of Hg (i.e., Hg^0^, Hg^II^, CH_3_Hg^I^ and (CH_3_)_2_Hg) play critical roles in the biogeochemical cycling of Hg. Hg^0^ allows for long‐range transport (Jackson 1997; Pirrone et al. 2009), Hg^II^ is the dominant reservoir for Hg in soils and aquatic systems (Fleck et al. 2015; Eklöf et al. 2018), and MMHg is bioconcentrated and biomagnified in aquatic food webs, reaching up to 80–100% of the total‐Hg (THg) measured in fish muscle (Bloom 1992; Mason et al. 2012; Bravo et al. 2014). As a consequence, MMHg exposure through fish consumption is of special concern for human health. A recent study performed in 175 countries, showed that 38% of studied populations (mainly insular and developing nations) were exposed to doses of MMHg above governmental thresholds (Lavoie et al. 2018). Indeed, concentration of Hg in fish is known to repeatedly overpass environmental quality guidelines even in absence of local sources (Depew et al. 2013; Åkerblom et al. 2014; Eagles‐Smith et al. 2016).

Comprehensive evaluations of the chemical and physical processes that govern Hg distribution and fate among the major environmental compartments can be found in the literature (Chételat et al. 2015; Sundseth et al. 2015; Kim et al. 2016; Bjørklund et al. 2017; Dranguet et al. 2017; Paranjape and Hall 2017; Klapstein and Driscoll 2018). Briefly, in the water column Hg^II^ can (1) be reduced to Hg^0^ and reemitted back to the atmosphere, (2) methylated to the organic form MMHg, or (3) bind to organic matter (OM) as well as inorganic particles and directly deposit to bottom sediments. MMHg formed in aquatic ecosystems can also deposit to sediments, be methylated and/or form DMHg. Part of DMHg might be re‐emitted to the atmosphere or again degraded to MMHg, which can also be biotically (Barkay et al. 2003) or abiotically demethylated (Fernández‐Gómez et al. 2013). Although some of the MMHg found in aquatic systems might come from the degradation of DMHg to MMHg, i.e., in oceans (Mason et al. 2012), several studies concluded that most of the MMHg measured in ecosystems was formed in situ or in the surrounding catchment (e.g., soils, wetlands, etc.) and subsequently transported into rivers, lakes (Louis et al. 1996; Eklöf et al. 2012; Bravo et al. 2017), and oceans (Schartup et al. 2015). Abiotic methylation of Hg^II^ is possible if suitable methyl donors are present (Celo et al. 2006; Munson et al. 2018). Nevertheless, recent studies have shown that biological Hg^II^ methylation is in most environments performed by a variety of microorganisms, carrying the *hgcA* and *hgcB* gene cluster (Gilmour et al. 2013, 2018; Parks et al. 2013; Yu et al. 2018). An increasing number of recent studies detailed below have intended to evaluate the biotic Hg^II^ methylation by studying the biodiversity and activity of *hgcAB*+ microorganisms (Gionfriddo et al. 2016; Bravo et al. 2018b,*a*; Bowman et al. 2019; Jones et al. 2019; Villar et al. 2019). Nevertheless, it is established that net MMHg production also depends on other concomitant processes, including (1) the composition and activity of the whole microbial community that in turn modulate the activity of *hgcAB*+ microorganisms (Bravo et al. 2018a), (2) physico‐chemistry that controls Hg^II^ bioavailability (Schaefer and Morel 2009; Jonsson et al. 2012; Chiasson‐Gould et al. 2014), and uptake in microorganisms (Schaefer et al. 2011), and (3) biotic and abiotic MMHg demethylation (Du et al. 2019).

Recent reviews critically summarized Hg^II^ uptake and MMHg efflux in methylating anaerobes, methods and equations to analyze Hg methylation rates (Regnell and Watras 2019) as well as chemotrophic and biotic Hg methylation and demethylation processes by anaerobes and phototrophs (Grégoire and Poulain 2018; Du et al. 2019). In this review, we aim to summarize the main findings on mechanisms responsible for the formation of MMHg, focusing in detail on the new knowledge recently gained on microorganisms involved in MMHg formation. We also aim to highlight pitfalls and limitations impeding the progress in the current understanding, and we propose a road map to overcome these limitations. Indeed, to improve our ability to predict MMHg generation in the environment, a current research priority is to better understand the distribution of methylating populations in the context of the physico‐chemical constraints known to affect MMHg production.

## *Methylmercury formation is widespread in the environment*

Recent advances in the methodology to (1) determine in situ Hg^II^ methylation (Jonsson et al. 2014) and (2) identify the organisms involved in this process (Parks et al. 2013; Christensen et al. 2016) have revealed that MMHg can be formed in a wider range of environments than previously identified. Indeed, 30 yr ago, first studies showed that Hg^II^ methylation in aquatic systems occurred mainly in sediments and under anaerobic conditions (Compeau and Bartha 1984; Korthals and Winfrey 1987; Pak and Bartha 1998). In general, sediments and sinking particles are a complex matrix of solid phases including clays, quartz, metal oxides (FeOOH, MnO_2_, AlO_3_) carbonates, sulfides and a number of other minerals and OM. They provide various microenvironments and habitats to organism populations notably bacteria, archaea, algae, diverse invertebrates, and so forth. To date, biological Hg^II^‐methylation is known to be mediated by species carrying the *hgcAB* gene cluster (Parks et al. 2013). Because all the identified microorganisms with Hg^II^‐methylating capacity were anaerobes, it was assumed for a long time that MMHg formation was occurring in strictly anoxic environments, i.e., sediments. Nonetheless, several studies revealed that Hg^II^‐methylation can occur in oxygen deficient zones of water column (Eckley et al. 2005; Malcolm et al. 2010), sediments (Drott et al. 2008; Hines et al. 2012; Bouchet et al. 2013; Jonsson et al. 2014; Bravo et al. 2015; Liem‐Nguyen et al. 2016) flooded soils, e.g., wetlands (Louis et al. 1996; Tjerngren et al. 2012; Windham‐Myers et al. 2014) and ponds (Lehnherr et al. 2012; MacMillan et al. 2015; Herrero Ortega et al. 2018). In recent years, Hg^II^‐methylation processes were in addition observed in microenvironments such as periphyton, growing on macrophytes (Cleckner et al. 1999; Mauro et al. 2002; Guimarães et al. 2006; Achá et al. 2011; Hamelin et al. 2011; Bouchet et al. 2018) and settling particles of oxic water columns, including pelagic ocean waters (Monperrus et al. 2007; Cossa et al. 2009; Sunderland et al. 2009;Lehnherr et al. 2011 ; Gascón Díez et al. 2016). Initially, MMHg formation in oxic waters was considered negligible due to high redox and low concentrations of bacteria and nutrients, but studies demonstrated that about 20–40% of the MMHg measured below the surface mixed layer originates from the surface and enters deeper ocean waters (Blum et al. 2013). Similarly, a query of more than 3500 publicly available microbial metagenomes performed by Podar et al. (2015) unveiled the presence of *hgcAB*‐like genes in sediments and in previously unsuspected environments, including invertebrate digestive tracts, thawing permafrost soils, coastal “dead zones,” soils and extreme environments. Moreover, a recent study assessing 243 metagenomes from the *Tara* Oceans expedition reported high abundances of *hgcAB* genes in 77 samples across all oceans (Villar et al. 2019). The progress in genetics (Gilmour et al. 2013; Parks et al. 2013; Podar et al. 2015; Bravo et al. 2018b,*a*; Liu et al. 2018b; Jones et al. 2019) combined with recent advances in the use of stable isotopes to determine Hg^II^ methylation rate constants in sediments (Monperrus et al. 2007; Jonsson et al. 2012; Bravo et al. 2014, 2015), lakes (Eckley and Hintelmann 2006), water columns and oceans (Munson et al. 2018) as well as in sinking particles of marine and lake waters (Lehnherr et al. 2011; Gascón Díez et al. 2016) have demonstrated that the potential for MMHg formation in the environment is widespread across ecosystems.

## *Toward a better understanding of microbial methylmercury formation*

### The discovery of *hgcAB*

Fifty years ago, a decade after the first observation of the Minamata disease in Japan, pioneering research pointed to surface sediments, and bacteria activity as responsible of Hg^II^‐methylation (Jensen and Jernelöv 1969). One of the first studies targeting MMHg and bacteria, evaluated the Hg^II^‐methylation capacity of *Pseudomonas fluorescens*, *Mycobaeterium phlei*, *Escherichia coli*, *Aerobacter aerogenes*, and *Bacillus megaterium* over a 7‐day period in pure cultures (Vonk and Sijpesteijn 1973). In the presence of sublethal amounts of HgCl_2_, tested bacteria produced 49 to 169 ng L^−1^ d^−1^ of MMHg in aerobic conditions (Vonk and Sijpesteijn 1973). Another decade later, sulfate‐reducing bacteria (SRB) were eventually identified as major Hg^II^‐methylator in saltmarsh through inhibition of their activity with sodium molybdate and isolation of *Desulfovibrio desulfuricans* from sediments (Compeau and Bartha 1985).

The relationship between bacterial sulfate reduction and Hg^II^‐methylation was studied adding Hg^II^ to anoxic sediment slurries or lake water overlying intact sediment cores collected in Quabbin Reservoir, MA (Gilmour et al. 1992). Comparable profiles of sulfate reduction and Hg^II^‐methylation in sediment cores were reported, further suggesting that Hg^II^‐methylation was linked to this specific bacterial metabolism. Almost 20 yr ago, a correlation between the Hg^II^‐methylation and sulfate reduction rates in sediment of a saltmarsh was also shown (King et al. 2000, 2001). Sulfate reduction was then accepted as the main metabolic pathway related to Hg^II^‐methylation. In 2006, two studies revealed the role of iron‐reducing bacteria (FeRB) on Hg^II^‐methylation in ferruginous conditions (Fleming et al. 2006; Kerin et al. 2006). In 2010, Hamelin et al. further identified methanogens as important Hg^II^‐methylators in lake periphyton. Two laboratory studies later confirmed the efficiency of methanogens in converting Hg^II^ to MMHg (Yu et al. 2013; Gilmour et al. 2018). By culturing and isolating Hg^II^‐methylating strains or by using inhibitors of known Hg^II^‐methylators such as molybdate for sulfate‐reduction and BESA for methanogenesis, Hg^II^‐methylation has mainly been then attributed to the action of SRB (Devereux et al. 1996; Pak and Bartha 1998; Hylander 2000; King et al. 2001; Achá et al. 2011, 2012; Yu et al. 2012; Bravo et al. 2016), and in some cases to FeRB (Fleming et al. 2006; Bravo et al. 2015, 2018b) as well as methanogens (Hamelin et al. 2011; Bravo et al. 2018a).

A recent breakthrough in the understanding of the biological Hg^II^‐methylation pathway was the identification of a two‐gene cluster, *hgcAB* ‐involved in C1 metabolism and the acetyl‐CoA pathway (Qian et al. 2016) required for Hg^II^‐methylation (Parks et al. 2013). The gene *hgcA* encodes a corrinoid protein that is essential for the biosynthesis of the folate branch of acetyl‐CoA pathway, whereas the gene *hgcB* encodes a ferredoxin‐like protein thought to be an electron donor to *hgcA* (Parks et al. 2013). Both provide methyl groups required for Hg^II^ methylation, although it is not clear whether MMHg production is a controlled or an accidental metabolic process (Qian et al. 2016). However, deletion of either gene eliminated Hg^II^ methylation in *Desulfovibrio desulfuricans* ND132 (Parks et al. 2013). By directly measuring Hg^II^ methylation in several bacterial and archaeal strains encoding *hgcAB*, Gilmour et al. (2013) confirmed that Hg^II^‐methylation capability could be predicted by the presence of *hgcAB* in the genome. For the first time, Gilmour et al. (2013) demonstrated Hg^II^‐methylation capability in previously completely unsuspected species including syntrophic, acetogenic, and fermentative *Firmicutes*.

In recent years, the biodiversity of *hgcAB* microorganisms was actively studied in contrasting environments. First biodiversity studies using this newly identified gene cluster were based on classical polymerase chain reaction (PCR) amplification with one pair of primers targeting *hgcA* developed based on the couple of available sequenced genomes of methylating strains at that time (Table 1), followed by cloning and sequencing (Bae et al. 2014; Liu et al. 2014; Schaefer et al. 2014; Smith et al. 2015). In soils of the Florida Everglades, the sequences identified were distributed in diverse phyla, including *Deltaproteobacteria*, *Chloroflexi, Firmicutes*, and *Methanomicrobia*; however, *hgcA* clone libraries from all sites were dominated by sequences clustering within the order *Syntrophobacterales* (Bae et al. 2014) (Table 2). By comparing the taxonomically identified *hgcA* sequences with the activity of SRB (mRNA of *dsrB* gene), Bae et al. concluded that *Syntrophobacterales* largely dominated the Hg^II^ methylating microbial community of the Florida Everglades (Bae et al. 2014). In the Three Gorges Reservoir in China, PCR amplification and sequencing of *hgcA* gene resulted in the identification of *δ‐Proteobacteria*, methanogens and a *Clostridia* group as putative Hg^II^ methylators in this ecosystem. Authors reported in addition a positive correlation between the abundance of *hgcA* and *dsrB* genes and MMHg concentrations, suggesting SRB as the main group responsible for Hg^II^ methylation in those systems (Luo et al. 2016). In Wanshan Hg mining area of China, the taxonomically annotated sequences were related to *δ‐Proteobacteria, Firmicutes, Chloroflexi*, and *Euryarchaeota* (Liu et al. 2014). In temperate and tropical wetland soils, *hgcA* gene sequences were attributed to *δ‐Proteobacteria*, *Chloroflexi*, and *Methanomicrobia* (Schaefer et al. 2014). In nine rice paddy soils sampled in three mining areas in China, *hgcA*+ microbes were dominated by *Proteobacteria* or *Euryarcheaota* in six and three sites, respectively. Only nine of the 190 operational taxonomic unit (OTUs) found in these rice paddy soils were common to all sites (Liu et al. 2018a).

**Table 1:** Published studies on *hgcA* diversity and quantification in environmental samples. Sequences and position of primers used, amplicon sizes, targets, and used methods are given.

References	Primer sequences 5′‐ > 3′	Target	Amplicon size (bp)	Methods
Bae et al. 2014	hgcA_268F GGNRTYAAY RTNTGGTGYGC	*hgcA‐hgcB*	888 to 945	PCR and cloning
	hgcB_1198R CADGCNCCRCAYTCVATRCA			
Bowman et al. 2019	NA			Metagenomics
Bravo et al. 2018a	As Schaefer et al. 2014			PCR and high throughput sequencing
Bravo et al. 2018b	As Schaefer et al. 2014			PCR and high throughput sequencing
Bravo et al. 2016	hgcA_262F GGNRTYAAYRTNTGGTGYGC	*hgcA*	650	qPCR
	hgcA_912R GGTGTAGGGGGTGCAGCCSGTRWARKT			
Christensen et al. 2016, 2018	ORNL‐HgcAB‐uni‐268F AAYGTCTGGTGYGCNGCVGG	*hgcA‐hgcB*	818 to 1020	PCR
	ORNL‐HgcAB‐uni‐1198R CABGCNCCRCAYTCCATRCA			
	ORNL‐Delta‐HgcA‐181F GCCAACTACAAGMTGASCTWC	*hgcA* of SRB	107	qPCR
	ORNL‐Delta‐HgcA‐287R CCSGCNGCRCACCAGACRTT			
	ORNL‐SRB‐firm‐HgcA‐444F TGGDCCGGTDARAGCWAARGATA	*hgcA* of Firmicutes	167	qPCR
	ORNL‐SRB‐firm‐HgcA‐610R AAAAGAGHAYBCCAAAAATCA			
	ORNL‐archaea‐HgcA‐184F AAYTAYWCNCTSAGYTTYGAYGC	*hgcA* of Archae	125	qPCR
	ORNL‐archaea‐HgcA‐308R TCDGTCCCRAABGTSCCYTT			
Dranguet et al. 2017	As Bravo et al. 2016			qPCR
Du et al. 2017	As Schaefer et al. 2014			qPCR, PCR, and cloning
Gionfriddo et al. 2016	NA			Metagenomics
Lei et al. 2019	As Christensen et al. 2016			qPCR
Liu et al. 2014	hgcA_626F GGNRTYAAYRTCTGGTGYGC	*hgcA*	315	PCR and cloning, qPCR
Liu et al. 2018a	hgcA_941R CGCATYTCCTTYTYBACNCC			
	As Schaefer et al. 2014 hgcA_515F GTGCCAGCMGCCGCGGTAA′ hgcA_806R GGACTACHVGGGTWTCTAAT	*hgcA*	291	PCR and cloning qPCR
Liu et al. 2018b	As Christensen et al. 2016	*hgcA* *hgcAB*		qPCR, metagenomics PacBio sequencing
Ma et al. 2017	As Bae et al. 2014			qPCR
Ndu et al. 2018	As Christensen et al. 2016			PCR and cloning, qPCR
Podar et al. 2015	NA	*hgcAB*		Metagenomics
Schaefer et al. 2014	hgcA_261F CGGCATCAAYGTCTGGTGYGC			
	hgcA_912R GGTGTAGGGGGTGCAGCCSGTRWARKT	*hgcA*		PCR and cloning
Villar et al. 2019	NA			Metagenomics
Vishnivetskaya et al. 2018	As Christensen et al. 2016			PCR and cloning, qPCR
Xu et al. 2019	As Schaefer et al. 2014			PCR and high throughput sequencing

**Table 2:** Main characteristics and outcomes of published studies on *hgcA* biodiversity in environmental samples.

Method	Studied environment	Number of sequence/reads	Number of OTUs	Dominant group	Dominant Deltaproteobacteria	Reference
PCR and cloning	Florida Everglades	220	168	*Deltaproteobacteria*	*Syntrophobacterales*	Bae et al. 2014
	Wetlands	108	40	*Methanomicrobia*	*Geobacter*	Schaefer et al. 2014
	Three gorges reservoir	151	151	Unidentified	Unidentified	Du et al. 2017
	Rice paddy soils	∼ 1800	190	*Proteobacteria* *Euryarchaeota*		Liu et al. 2018a
	Laboratory and sediment slurries			Unidentified	Unidentified	Ndu et al. 2018
	Rice paddy soils	5	?	Unidentified		Vishnivetskaya et al. 2018
	Sulfate‐impacted lakes	300	174	*Geobacteraceae* *Methanomicrobia* *Unidentified*		Jones et al. 2019
PCR and high throughput sequencing	Boreal lakes	78,642	225	*Deltaproteobacteria*	Unidentified	Bravo et al. 2018a
	Lake Geneva	741,890	356	*Deltaproteobacteria*	Unidentified (*Geobacter*)	Bravo et al. 2018b
	Rice paddy soils					Liu et al. 2018a
	Boreal forest soils	1,257,577	573	*Deltaproteobacteria*	Unidentified (*Geobacter*)	Xu et al. 2019
Metagenomics	Global	823,000,000		Environmental compartment dependent		Podar et al. 2015
	Antarctic Sea‐ice and brine			*Nitrospinae*		Gionfriddo et al. 2016
	Rice paddy soils	901,610,484		*Methanoregula* spp.	*Geobacter*	Liu et al. 2018b
	Sulfate‐impacted lakes	885,923	27	*Deltaproteobacteria*		Jones et al. 2019
	Eight sites	381–102 1		*Deltaproteobacteria*		Christensen et al. 2019
	Tara gene catalogs oceans	111,530,851	10	*Nitrospinae*	*Desulfovibrionales*	Villar et al. 2019
Metaproteomics	EFPC and Hinds Creek, TN	15,270–16,852		*Deltaproteobacteria*		Christensen et al. 2019

Based on a higher number of sequenced microbial genomes now available, Christensen et al. (2016) developed a broad range *hgcAB* primer pair that improved the coverage of prior developed primers by 10% (Bae et al. 2014; Schaefer et al. 2014), and several clade‐specific PCR primers to improve amplification of the various members of the Hg^II^‐methylating community (Christensen et al. 2016) (Table 1). In sediments, these clade‐specific primers were useful to detect Hg^II^‐methylating *δ‐Proteobacteria* and *Archaea* but failed to detect Hg^II^‐methylating *Firmicutes* (Christensen et al. 2017).

Recent studies based on *hgcAB* biodiversity analyzed both *hgcA* (using one single pair of primers, Table 1) and 16S rRNA genes by high‐throughput illumina sequencing techniques, allowing a deeper sequencing than cloning‐sequencing and hence resulting in a higher number of OTUs. Results evidenced that microbial Hg^II^‐methylating community was composed of members of various clades, including SRB, FeRB, methanogens and syntrophs in temperate and boreal lake sediments (Bravo et al. 2018a) as well as in boreal forest soils (Xu et al. 2019). In boreal lakes, besides the identification of Hg^II^‐methylating methanogens and *Geobacteraceae*, authors further showed thanks to inhibition of sulfate reduction with molybdate that only 40% of MMHg was dependent on SRB (Bravo et al. 2018a). Another study performed in sediments impacted by a sewage treatment plant showed that Hg^II^ methylating *Geobacteraceae* seemed to have an important role in Hg^II^ methylation in sediments showing ferruginous conditions (Bravo et al. 2018b). Importantly, those studies suggested that the differences in the distributions of Hg^II^ methylating taxa among the different sites might derive primarily from different species of the same family having different niche requirements (Bravo et al. 2018a). In particular, the high relative abundance of phytoplankton‐derived OM and the presence of specific strains of non‐Hg^II^‐methylating bacteria involved in OM decomposition (e.g., *Rhizobiales*, *Fibrobacterales*, *Holophalages*, etc.) seems to be essential in creating a niche that promotes Hg^II^ methylation (Bravo et al. 2018a; Lei et al. 2019). Another study conducted in sulfate‐impacted lakes combined cloning‐sequencing of *hgcA* with metagenomics targeting *hgcA* gene and genes involved in other metabolic functions (Jones et al. 2019). This approach yielded after in silico assembly of reads with overlapping sequences in a relatively low number of contigs (27), but revealed a high occurrence of *hgcA* genes together with genes involved in sulfate‐reduction and fermentation, but also that some abundant *hgcA*+ microbes were related to uncultivated microbes, such as *Aminicenantes*, *Kiritimatiellaeota*, *Spirochaetes*, as well as completely unidentified microbes. Data showed that potential methylators from uncultivated organisms occurred more abundantly than previously anticipated in these overlooked clades and that they can dominate the methylating community in certain circumstances (Jones et al. 2019).

A recent study conducted in rice paddy soils combining metagenomics illumina sequencing and long‐read PacBio sequencing, which allows overcoming the inherent risk of short‐reads chimeric assembly, revealed the dominance of *Geobacter* spp. for bacteria and *Methanoregula* spp. for *Archaea* (Liu et al. 2018b). These authors hypothesize a syntrophic interaction between both species and in addition reported a significant correlation between *Geobacter hgcA*+ DNA relative abundance and MMHg concentration in soils (Liu et al. 2018b), supporting an important role of this genus for MMHg production in iron‐rich paddy soils. A recent study in sediments collected in eutrophic lakes showing cyanobacteria blooms in China also reported a correlation between *Archae hgcA*+ DNA relative abundance and MMHg concentration (Lei et al. 2019). However, several studies that tried to correlate the level of expression of *hgcA* mRNA with Hg^II^ methylation rates (Goñi‐Urriza et al. 2015; Bravo et al. 2016; Christensen et al. 2019) were mostly unsuccessful. For example, in pure cultures of *Desulfovibrio dechloroacetivorans* BerOc1, the level of expression of *hgcA* was not correlated with Hg^II^ methylation rates (Goñi‐Urriza et al. 2015). Similarity, in sediments collected in a river impacted by effluents from a chlor‐alkali plant, data suggested that physico‐chemistry varied significantly among reservoirs, while functional gene activities, including *hgcA*, were very similar and did not correlate with MMHg concentrations (Bravo et al. 2016). In contrast, in Hg‐contaminated paddy soils, the *hgcAB* copy number increased with both increasing THg and MMHg concentrations (Vishnivetskaya et al. 2018).

Despite the differences in primer pairs used in the studies mentioned earlier (Table 1), until now, data globally suggested that in *hgcA* ^*+*^ *δ‐Proteobacteria* communities are abundant in surface sediments, but in some sites *hgcA* ^*+*^ methanogens and other *hgcA* ^*+*^ uncultivated groups are prevalent (Christensen et al. 2017, 2019; Vishnivetskaya et al. 2018; Bravo et al. 2018a; Jones et al. 2019). Among *δ‐Proteobacteria*, syntrophs and *Geobacter* spp. appear more prevalent in *hgcA* ^+^ community than previously expected. Moreover, other groups of *hgcA* ^−^ microbes seem to be of high importance for Hg^II^‐methylating species, certainly by providing some kind of dependence or mutualistic relationship in sediments (Bravo et al. 2018a; Liu et al. 2018b). For example, syntrophs have been shown to modulate Hg^II^ methylation of *hgcA* strains in controlled exposures (Yu et al. 2018). Syntrophy between methanogens or propionate utilizing syntrophs and SRB is hypothesized to enhance methylation in environments devoid of sulfate or where the type and concentration of energy sources are limiting.

Studies listed above focused in freshwaters and therefore the microorganisms processing Hg^II^ to MMHg in the ocean are still barely described. Podar et al. (2015) showed that *hgcAB* appeared to be abundant in marine sediments but they rarely found it in pelagic marine water column, as from 138 metagenome samples analyzed, only seven showed evidence of *hgcAB*. A recent analysis of 243 seawater metagenome samples from 68 different sites of the *Tara* Oceans revealed high abundances of *hgcAB* corresponding to taxonomic relatives of known Hg^II^ methylators from *Deltaproteobacteria*, *Firmicutes*, and *Chloroflexi* across all oceans, with the exception of the Arctic that was not studied (Villar et al. 2019). More recently, Bowman et al. (2019), combining PCR amplification and shotgun metagenomics, searched the *hgcAB* gene cluster in Arctic Ocean seawater without success. Out of all the *hgcA*‐like genes found in the queries of marine metagenomes, the *Nitrospina* phylum, a marine nitrite oxidizing bacteria abundant in oxygen‐deficient zones, appeared to be widespread, predominant and likely a key player for MMHg production in the oxic subsurface waters of the global ocean (Villar et al. 2019), including the Arctic (Bowman et al. 2019) as well as Antarctic sea ice–brine–sea water interfaces (Gionfriddo et al. 2016). However, despite metagenomic evidence for the abundance of *Nitrospina* in the global ocean, the few cultured strains harboring a fused *hgcAB‐*like gene (*Methanococcoides methylutens* and *Pyrococcus furiosus*) were unable to produce MMHg in experimental conditions (Podar et al. 2015; Gilmour et al. 2018). Moreover, there is yet no experimental or observational report on the expression of *hgcAB*‐like genes in *Nitrospina* bacteria. As such, an experimental evidence of the Hg^II^ methylating capacity in *Nitrospina* is awaited to confirm their role as important Hg^II^ methylators in the global ocean. As MMHg has been detected in the water column of every ocean basin, except for the Indian Ocean (Bowman et al. 2019), it is crucial to unveil the role of the microorganisms involved in both MMHg and degradation in seawaters.

These results and observations reveal the diversity of Hg^II^‐methylating microbial communities’ structure across ecosystems and point for the need of a thorough investigation of their functioning. Notably further work is necessary to better understand the contribution of overlooked microbial groups in Hg^II^ methylation, highlighted by the high proportion of unidentified OTUs found in recent studies concerning *hgcAB* biodiversity (Table 2). However, it would be useful to agree on a standardized protocol to conduct *hgcAB* biodiversity studies and have an *hgcAB* open‐access library, as published studies are currently difficult to directly compare due to differences in methods, including primer pairs, alignment algorithms used and depth of sequencing.

### Hg^II^ methylators are part of a complex microbial community

As described in the previous sections, current knowledge established that MMHg net production was linked to biotic and abiotic variables. Microorganisms behave differently from one system to another due to interactions with the physico‐chemical variables but also with other organisms of their (micro)environment (Andersson et al. 2014; Bravo et al. 2018a). Studies with one strain can only describe the metabolism of this strain in a batch (Andersson et al. 2014), which is useful for a mechanistic understanding of its potential metabolism. However, it cannot be straightforwardly applied for environmental predictions because its metabolism is likely modified by the activity of other microbial groups and the ambient physico‐chemistry. In this sense, one of the most insightful discoveries is the syntrophic Hg^II^ methylation recently described in both laboratory (Kerin et al. 2006; Ranchou‐Peyruse et al. 2009) and field studies (Yu et al. 2018). Syntrophy is just a “proof of concept” illustrating the complexity of microbial communities carrying out Hg^II^ methylation. It is also important to consider that within a microbial community, besides Hg^II^, some bacteria carrying out the *merB* gene or other genes yet to be discovered, might demethylate MMHg (Barkay et al. 2003).

Electron donors are also essential for Hg^II^ methylation. Different microbial clades are involved in the anaerobic oxidation of OM from complex organic compounds generally that goes through several steps and processes (Gilmour et al. 2013; Bae et al. 2014). For example, an initial hydrolysis of large organic substances is followed by a fermentation of intermediates into smaller organic molecules, such as lactate, propionate, butyrate, acetate, and formate, as well as CO_2_ and H_2_. These fermentation products might then be used as electron donors for *Geobacterales, Desulfovibrionales*, and *Syntrophobacterales* known to host Hg^II^ methylators. Hg^II^ methylators thus likely rely on other microorganisms involved in the degradation of large organic compounds. Furthermore, it was also demonstrated that the specific metabolism of one strain may provoke a new metabolism (unknown) in another strain; this is called the Quorum sensing (Lovley and Chapelle 1995). It is therefore very important to better tackle the complexity of microbial communities and describe the compendium of metabolic processes that can affect directly or indirectly Hg^II^ methylation.

## *Physico‐chemistry plays a pivotal role in Hg^II^ methylation*

**Figure 1:**
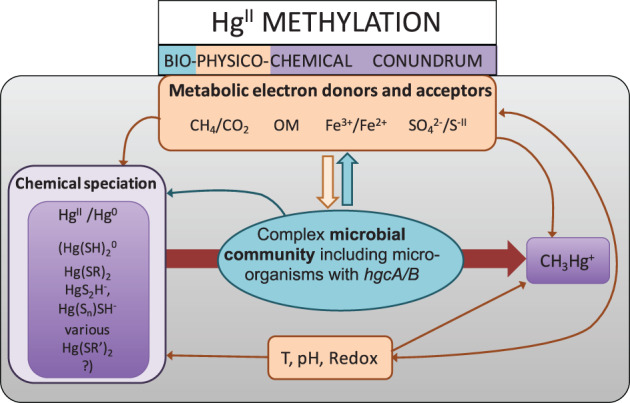
Conceptual summary of the biological and chemical interplays affecting Hg^II^ methylation in the environment. Orange boxes and arrows refer to geochemical variables directly affecting microbial activity and Hg speciation. Purple boxes refer to Hg chemical forms. The red arrow indicates the transformation of Hg^II^ to CH_3_Hg^+^. Blue refers to a compendium of metabolic processes occurring in the environment, among these processes Hg^II^ methylation carried out by the *hgcAB* gene cluster.

Besides the presence and diversity of Hg^II^ methylating microbes, Hg^II^ methylation depends on the amount of Hg^II^ bioavailable for methylation (Schaefer et al. 2011; Jonsson et al. 2014), which is determined by chemical speciation of Hg^II^, solubility of the Hg‐S particles (Hsu‐Kim et al. 2013; Liem‐Nguyen et al. 2017) as well as the availability of electron donors and acceptors for Hg^II^ methylating microorganisms (Desrochers et al. 2015). Hg^II^ methylation is a bio‐physico‐chemical conundrum because both the amount of Hg^II^ available for methylation and the activity of microorganisms involved in the process are determined by multiple physico‐chemical variables such as sulfur (Skyllberg et al. 2003; Drott et al. 2007), iron (Bravo et al. 2015) and OM concentration and speciation (Schartup et al. 2013; Bravo et al. 2017) as well as Eh, pH, nutrient availability, and temperature (Ullrich et al. 2001; Paranjape and Hall 2017) (Fig. 1).

### Variables affecting Hg^II^ chemical speciation

Salinity, sulfur, iron, and OM affect Hg^II^ chemical speciation. Sea salt anions may also affect Hg^II^ speciation and/or methylation in estuarine and marine environments. In marine waters, Hg^II^ forms compounds with chlorine (HgCl_3_^−^ and HgCl_4_^2−^) to a greater extent than oxides, that are in turn formed in freshwaters (Mason and Fitzgerald 1993). A lower Hg^II^ methylating activity in marine and estuarine sediments than in freshwater sediments has been attributed to the formation of charged chloride but also sulfide complexes, that undergo a slower methylation processes than other Hg^II^ forms (Gårdfeldt et al. 2003; Jonsson et al. 2012; Gworek et al. 2016).

In the context of the biological and chemical interplays controlling Hg^II^ methylation, sulfur plays a central role by directly affecting Hg^II^ speciation and solubility (Jonsson et al. 2012; Graham et al. 2013; Hsu‐Kim et al. 2013; Liem‐Nguyen et al. 2017) and consequently its bioavailability (Schaefer and Morel 2009; Chiasson‐Gould et al. 2014; Schartup et al. 2015; Mazrui et al. 2016). Reactions between Hg^II^ and sulfide control the formation of the solid phase metacinnabar, β‐HgS(s) but also aqueous complexes such as Hg(SH)_2_^0^, HgS_2_H^−^, and HgS_2_^2−^ or polysulfides HgS_n_SH−(aq) (*n* = 4–6) (Liem‐Nguyen et al. 2017). Elevated sulfide concentrations, eventually limits Hg^II^ bioavailability for methylation (Drott et al. 2007; Hsu‐Kim et al. 2013; Bigham et al. 2017). Conversely low sulfide concentrations might enhance Hg^II^ methylation processes (Benoit et al. 1999; Graham et al. 2012). Natural OM often contain thiols, which sulfhydryl group has a high capacity to complex Hg^II^ and MMHg (Skyllberg et al. 2006; Skyllberg 2008). Iron plays also a key role on Hg^II^ methylation as its reduced form Fe^2+^ can scavenge sulfide, form stable iron–sulfur compounds (FeS, Fe_3_S_4_, or FeS_2_) and then might let Hg^II^ bioavailable for methylation (i.e., ferruginous conditions; Bravo et al. 2015). Each of these Hg^II^ complexes, i.e., inorganic sulfides, polysulfides, and OM in aqueous, solid, and adsorbed phases, show different reactivity in the environment. For example, Hg^II^ methylation rate constants in estuarine sediments spanned over two orders of magnitude depending on the chemical form: metacinnabar (β—HgS(s)) < cinnabar (α—HgS(s)) < Hg^II^ reacted with mackinawite (≡FeS‐Hg^II^) < Hg^II^ bonded to natural OM (NOM—Hg^II^) < (Hg(NO_3_)_2_(aq)) that is a typical aqueous tracer (Jonsson et al. 2012).

The effect of natural OM on the sediment and pore water‐partitioning coefficient for Hg^II^ (Hammerschmidt et al. 2008; Liem‐Nguyen et al. 2016) needs still to be determined. Under low OM and porewater sulfide concentrations, Hg^II^ partitioning coefficient becomes a major factor for methylation (Mitchell and Gilmour 2008; Hollweg et al. 2009). Some studies have shown that increases in natural OM concentrations might also raise partitioning coefficients in sediment and pore water and have shown to decrease Hg^II^ concentration in the pore water, and thus its bioavailability for uptake by methylating microorganisms (Liem‐Nguyen et al. 2016). In contrast, other studies concluded that OM content did not explain variations in Hg^II^ partitioning, most likely only a limited fraction of OM was relevant for Hg complexation or because it is also possible that the composition, rather than amount, of OM controls Hg^II^ partitioning (Schartup et al. 2013).

High concentrations of OM decreased Hg^II^ bioavailability in laboratory experiments (Chiasson‐Gould et al. 2014) and in marine sediments (Hammerschmidt et al. 2008). Moreover, OM might also be important in determining Hg^II^ bioavailability by stabilizing HgS particles at nanoscale that can be methylated by anaerobic bacteria (Graham et al. 2013; Hsu‐Kim et al. 2013). To progress further in the understanding of Hg^II^ methylation in future research, it is of the upmost importance to measure and model Hg^II^ chemical speciation, which is ultimately determining the Hg^II^ availability for methylating bacteria (Fig. 1, violet box).

### Variables affecting microbial activity

Sulfur, iron, OM, redox, pH, nutrients, and temperature affect microbial activity. Both sulfur and iron have oxidized forms (SO_4_^2−^ and Fe^3+^, respectively) that can serve as electron acceptor for some Hg^II^ methylating bacteria (e.g., SRB and FeRB). Natural OM, besides its strong capacity to bind Hg^II^ and influence Hg^II^ speciation, controls microbial activity and Hg^II^ methylation (Graham et al. 2013; Hsu‐Kim et al. 2013). The molecular composition of OM shows a central role in controlling Hg^II^ methylation (Bravo et al. 2017), notably phytoplankton derived OM and fresh humic matter were associated with both high bacterial activity and Hg^II^ methylation rates in sediments (Graham et al. 2013; Schartup et al. 2013; Mazrui et al. 2016; Bravo et al. 2017; Christensen et al. 2017; Herrero Ortega et al. 2018). Also, an increase in nutrients, associated to an enhanced algae biomass production, increased MMHg formation in sediments (Bravo et al. 2017; Herrero Ortega et al. 2018). Changes in redox conditions might also affect iron, sulfur and Hg^II^ speciation (Liem‐Nguyen et al. 2016), and the activity of the microorganisms (Grégoire and Poulain 2018) and consequently Hg^II^ methylation (Fig. 1, orange boxes and arrows). Furthermore, Hg^II^ methylation strongly depends on the activity of the whole microbial community (Weber et al. 2006; DeAngelis et al. 2010). Therefore, all the physico‐chemical variables causing increased microbial activity in sediments, such as temperature (Gudasz et al. 2010), might indirectly lead to enhanced Hg^II^ methylation (Bravo et al. 2017; Dijkstra et al. 2011).

From all the studies mentioned earlier, we can conclude that both microbial activity and Hg^II^ chemical speciation control the formation of MMHg in the environment. Therefore, Hg cycling and in particular, the methylation of Hg^II^ is an intricate process regulated by both physico‐chemical and biological constrains that needs holistic approaches to be fully understood.

## *Knowledge gaps and uncertainties*

### A high number of unidentified putative Hg^II^ methylators

The complexity of microbial communities and its implications for Hg cycling is one of the main current challenges. First biodiversity studies used 16S rRNA gene to investigate the diversity of Hg^II^ methylating microbial communities. But biodiversity of this gene cannot provide reliable and robust identification of Hg^II^ methylator diversity and abundance, because *hgcAB+* strains are too rare (<1%) and not well identified in 16S rRNA databases (<300 species) (Miller and Bassler 2001; Christensen et al. 2019). Recent studies targeting *hgcAB* are interesting, but these molecular approaches entail several limits. First, as for all microbial biodiversity studies, the DNA extraction protocols need to be well planned to ensure clean subsampling avoiding contamination and may need to be optimized for the efficient recovery of DNA and the elimination of potential inhibitors for PCR and/or sequencing technologies. Indeed, DNA extraction and PCR amplification are known to be prone to artifacts due to any combination of high primer mismatch, low abundance, and/or low DNA extraction efficiency that can significantly affect results (Bravo et al. 2018a; Epp et al. 2019). Although claimed as “universal,” primers inherently show preferences and limitations in covering all species equally among different environmental samples. More specifically, currently available primer pairs for the gene *hgcA* are predicted to cover 84% (Schaefer et al. 2014) and 94% (Christensen et al. 2016) of the whole biodiversity by PCR‐based approaches. However, within amplified sequences of *hgcA* a significant proportion cannot be identified above the clade level because of (1) the lack of identified organisms in the databases, and (2) the low conservation of *hgcA* gene that is not ideal for biodiversity studies. Consequently, a significant proportion of OTUs are attributed to unidentified species. Indeed, identification of *hgcA* sequences from short reads is made by aligning metagenomics data with a set of known gene sequences isolated from cultivated strains. This approach is prone to inaccuracies, especially if the data are evaluated down to the genus level. For example, in recent studies 62% (Bravo et al. 2018b) and 57% (Bravo et al. 2018a) of the identified OTUs could be taxonomically assigned at the order level. More in detail, different algorithms are available to identify OTUs e.g., simple sequence alignment‐based algorithms (e.g., Basic Local Alignment Search Tool [BLAST]) or profile hidden Markov model (HMM)‐based searches, which are expected to be more sensitive and accurate in identifying homologs. Nonetheless, the HMM model is satisfying for *hgcA* but not for *hgcB* that cannot be confidently differentiates from other ferredoxin‐encoding genes due to homology (Christensen et al. 2019). Besides, both primers and algorithms are developed on sequences from cultivated strains and consequently identify those species with a higher efficiency (Podar et al. 2015; Bravo et al. 2018b; Liu et al. 2018b). Moreover, only few hundred sequenced genomes of methylating strains are currently available in databases. Although this number is increasing regularly, databases still do not include reliably microorganisms that cannot be grown in the laboratory. Besides we do not have information on the rates and methylation capabilities of these uncultivated populations and thus we cannot affirm that the genes identified code for MMHg production in these organisms. Further, we cannot evaluate how Hg methylation rates in these organisms compare to other methylators in culture. In sum, available sequences are far from being sufficient to confidently identify all OTUs homologs of *hgcA* and might not allow identifying unknown microbes. This is a current inherent technical limit that has to be considered when interpreting data.

However, studies conducted on DNA do not reflect the activity of the protein or the enzyme preforming the process, but only the possibility that a strain could sometime methylate Hg^II^. Although the use of RNA should theoretically provide more detailed information on the activity of the organisms involved in Hg^II^, up to now the abundance of *hgcAB* transcripts could not be correlated to Hg^II^ methylation or MMHg concentration (Goñi‐Urriza et al. 2015; Bravo et al. 2016; Vishnivetskaya et al. 2018; Christensen et al. 2019), except in one study (Ledeker and De Long 2013). The precise factors regulating *hgcA* gene and protein expression are not identified yet but it seems that *hgcA* gene could be constitutively expressed or regulated by carbon, metabolism, but Hg does not appear to be a key regulator (Goñi‐Urriza et al. 2015; Christensen et al. 2019). Moreover, the correlation between mRNA and protein abundance is not necessarily linear as posttranslational regulations can take place. In addition to the factors regulating *hgcA* gene expression, it is important to consider that net MMHg production also depends on MMHg demethylation activity. Therefore, the absence of correlation between *hgcA* gene and MMHg concentration is not surprising and highlights the complexity of predicting MMHg concentration dynamics in the environment.

Currently, the scientific community lacks a straightforward method to directly measure the activity of *hgcAB* at the protein level. New metaproteomic approaches are currently developed and could in the near future allow a direct analysis of hgcA protein abundance in the environment (Meier et al. 2019), but this approach has been tested only once to study *hgcA* biodiversity yet (Christensen et al. 2019). Metagenomics, metatranscriptomics, and metaproteomics offer a deeper sequencing and a wealth of information than earlier but they are cost and time‐consuming to conduct analysis of hundreds of samples. This highlights that we still lack of a simple and accurate method to identify Hg^II^‐methylating microbial activity in environmental samples.

Another issue is that there is currently no medium or protocol to reliably culture a whole microbial community including FeRB, SRB, firmicutes, *Clostridia*, and so forth. Even studies with intact sediments in controlled experimental conditions can only approximate interactions of microbial community in situ and need to be confirmed in the field. For this reason, it is difficult to study and explain interactions of microbial communities through controlled experiments using a single strain. For example, microbial strains showing maximum methylation rates in the laboratory may not be as active in complex consortia under real field conditions. Metagenomics, metatranscriptomics, and metaproteomics could help to progress in the elucidation of the biological metabolism behind Hg^II^ methylation in the field. Instead of identifying different taxa, these approaches combine genes coding for all metabolic pathways of the different taxa to describe the potential function of the whole community (metagenomics) or its activity in one compartment (sediment, water, soil, etc.) at the moment of the sampling (metatranscriptomics and metaproteomics). Nonetheless, available metagenomics approaches have seldom identified *hgcA* sequences (i.e., 63 out of 203 metagenome projects revealed *hgcA* occurrence; Podar et al. 2015; and 77 out 243, Villar et al. 2019) supporting that new study should be conducted in environments relevant for Hg^II^ methylation. Furthermore, metatranscriptomics and metaproteomics are still rarely applied, most likely because they are expensive and time‐consuming. However, although we believe that these approaches are promising and their use will increase in coming years, they will need to be coupled with a detailed analysis of environmental conditions (e.g., physico‐chemistry analysis) to be fully informative.

### Are we measuring realistic Hg^II^ methylation rates?

Last but not least, besides limitations to directly measure biological process responsible for Hg^II^ methylation, there are several pitfalls on the methods used to determine Hg^II^ methylation at environmentally relevant Hg^II^ concentrations. Several approaches have been used to estimate MMHg formation: using labeled Hg^II^ forms with radio‐isotopes (Goñi‐Urriza et al. 2015; Bravo et al. 2016), stable isotopes (Ramlal et al. 1986), by measuring the percentage of total Hg and MMHg (%MMHg) (Hintelmann et al. 2000) or the change in MMHg concentration over time (Drott et al. 2008). The amendment of standards enriched in Hg^II^ stable isotope tracers to environmental samples is now widely used in different laboratories because they allow determining Hg^II^ methylation rates and MMHg demethylation rates simultaneously. However, it is known that the geochemical form of the Hg^II^ isotope used as tracer determines its reactivity (i.e., methylation rate) in the environment. For example, Hg^II^ methylation rate constants in estuarine sediments spanned over two orders of magnitude from metacinnabar to a typical aqueous tracer such as Hg(NO_3_)_2_(aq) (Jonsson et al. 2012). Therefore, Hg^II^ methylation rate constants measured using a highly available tracer might result in an overestimation of the in situ Hg^II^ methylation rate, when the tracer is a poor representative of the indigenous Hg^II^ chemical forms. The %MMHg and the change of MMHg concentration overtime represent the net MMHg, accounting for MMHg degradation processes and while it might be useful to predict MMHg concentrations in the environment, its use is limited to provide mechanistic understanding of Hg^II^ methylation processes.

From this literature review, we observe that after 50 yr of efforts to study Hg^II^ methylation through the world, knowledge has greatly increased, but there are still many aspects within the bio‐physico‐chemistry of MMHg formation that need to be unveiled in the natural environment because: (1) there is a lack of techniques and methods for the measurements of the different Hg^II^ chemical species available for methylation; (2) the physico‐chemical factors affecting Hg^II^ chemistry are still only partly understood; (3) the biological mechanisms involved in the whole bio‐physico‐chemical process of MMHg net production remains to be understood and described more accurately.

## *How to study methylmercury formation in future research?*

Besides current limits inherent to used analysis described earlier, main questions lacking a clear answer concerning the behavior of Hg in environmental systems include: What are all the Hg^II^ chemical species available for methylation? How diverse are *hgcA* ^+^ species? How does microbial community consortium impact the amount and speciation of Hg^II^ available for methylation? And what is the impact of the *hgcA* ^−^ microbial species on the activity of Hg^II^ methylators? Do techniques exist to tackle these questions?

**Figure 2:**
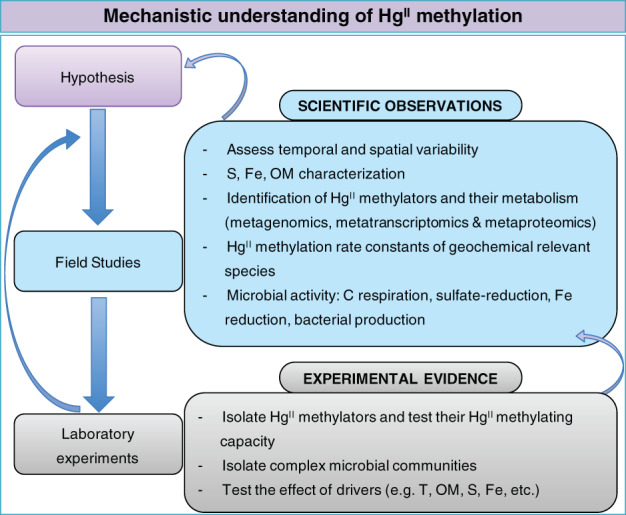
Schematic representation of the proposed conceptual iterative strategy for studying Hg^II^ methylation.

Laboratory studies are essential to identify mechanisms driving Hg^II^ methylation but need to include more realistic scenarios and/or be validated under field conditions. Currently, in situ studies to elucidate the biodiversity of Hg^II^ methylators and determine the drivers of their activity need to be conducted more widely and in more diverse environments. For in situ studies, Hg^II^ speciation and Hg^II^ rates need to be determined, and accompanied by the study of the factors controlling the activity of Hg^II^ methylating microorganisms. Determination of the methylating activity of natural microbial assemblages in relation to sediment characteristics, specific environmental conditions and the level of Hg contamination needs to be undertaken to validate laboratory‐based measurements and to improve our understanding of Hg cycling in contaminated environments (Fig. 2). In this context, interactions between microbial communities and the physico‐chemistry are key to predict Hg^II^ methylation as several studies pointed out that physico‐chemistry rather than the microbial community structure were determining Hg^II^ methylation rate constants in lake sediments (Bravo et al. 2016, 2017, 2018a; Liu et al. 2018b). This is likely true but it should not be forgotten that the local physico‐chemistry is also the result of the microbial activity (Bravo et al. 2017, 2018b).

**Figure 3:**
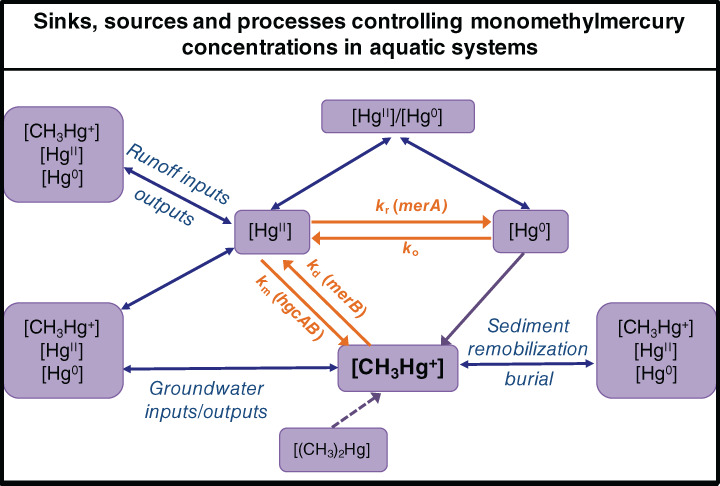
Proposed model to quantify MMHg in freshwater systems. The model considers formation (*k*_m_, Hg^II^ methylation) and degradation (*k*_d_, MMHg demethylation) and the *inputs* and *outputs* of MMHg. Processes are represented with orange arrows, known functional genes are shown in brackets. Transport of MMHg to the system or out the system is represented by blue arrows.

We can use existing and future new molecular biology and chemistry techniques to study Hg^II^ methylation, but studies have now to put more effort on combining metagenomics, metranscriptomics, and metaproteomics approaches with experiments on bacterial isolates and also complex matrices (i.e., sediments, marine waters, etc.) to explain more in detail environmental mechanisms (Fig. 2). Last, we should keep in mind that MMHg in aquatic systems is the net result of different processes: (1) formation (Hg^II^ methylation), (2) degradation (MMHg demethylation), and (3) the inputs and outputs of the system (Fig. 3). Therefore, when studying Hg^II^ methylation, we target only a limited part of the whole Hg cycling determining MMHg concentrations in the environment. Future studies need to be invested in studying concomitantly the drivers of MMHg methylation and MMHg demethylation mechanisms in situ as well as the transport of MMHg within the aquatic network, which is indeed highly controlled by hydrological processes (Fig. 3).

## Conflict of Interest

None declared.
